# Global Longitudinal Strain or Measurement of Ejection Fraction: Which Method is Better in Stratifying Patients with Heart Failure?

**DOI:** 10.5935/abc.20190151

**Published:** 2019-08

**Authors:** Filipe Ferrari, Willian Roberto Menegazzo

**Affiliations:** 1 Graduate Program in Cardiology and Cardiovascular Sciences, Hospital de Clínicas de Porto Alegre (HCPA), Universidade Federal do Rio Grande do Sul, Porto Alegre, RS - Brazil; 2 Exercise Cardiology Research Group (CardioEx), Hospital de Clínicas de Porto Alegre, Universidade Federal do Rio Grande do Sul, Porto Alegre, RS - Brazil

**Keywords:** Heart Failure, Systolic, Myocardial Contraction, Myocardial Stunning, Stroke Volume: Strain, Echocardiography/methods

Heart failure (HF) is a complex syndrome that has a poor prognosis and a stigma of high mortality.^1^ The current prevalence estimated in the United States is six million cases, with a predicted incidence of another two million patients until 2030.^2^ Brazil, specifically, had more than 26 thousand deaths by HF in 2012 and approximately 230 thousand hospitalizations attributed to this disease.^3^

The main HF symptoms include progressive dyspnea, fatigue, exercise intolerance, and signs of volume overload, reducing the functional capacity and quality of life of patients and greatly increasing the risk of morbidity and mortality.^4^ In this regard, a peak oxygen consumption (maxVO_2_), on average, approximately 50% lower is not uncommon in HF patients when compared to healthy individuals paired by variables such as age and gender.^5^ The cardiopulmonary exercise testing (CPET) is a method widely used and trusted in this scenario, with a consistent role in risk stratification of HF patients and various variables obtained with consolidated prognostic value. MaxVO_2_ is an important marker of one-year mortality, surpassing ejection fraction and pulmonary capillary wedge pressure, used as Class I to define candidates for heart transplantation.^6^ Other prognostic markers obtained from CPET that proved to be important in this population include the measurement of ventilatory efficiency through the VE/VCO_2_ slope, regular ventilation, oxygen uptake efficiency slope (OUES), heart rate recovery (HRR) in the first minute, chronotropic competence, and partial pressure of carbon dioxide at rest (PetCO_2_).^7,8^

HF patients are usually classified according to their left ventricular ejection fraction (LVEF); however, the prognostic value of LVEF can be controversial.^9^ Following this reasoning, although the LVEF measurement is a validated method that has been widely used for decades, the assessment of myocardial deformation with the Global Longitudinal Strain (GLS) has shown greater effectiveness in analyzing the overall breakdown of the left ventricle when compared to the LVEF measurement. GLS can provide an additional value for prognostic HF stratification, regardless of the LVEF values, and serve as an auxiliary instrument for therapeutic decision making in specific clinical situations in this population, such as: cardiac defibrillator and resynchronization device implantation, indication of ventricular assist devices, and follow-up of patients with cardiotoxicity due to chemotherapeutic agents.^10^

Recently, Park et al.^11^ assessed the prognostic value of GLS in more than 4 thousand individuals with acute HF, divided into preserved (≥50%), mid-range (40-49%), and reduced LVEF (<40%). The primary outcome analyzed was all-cause mortality, evaluated over five years. Patients with reduced and preserved LVEF presented lower and higher GLS, respectively. GLS, but not LVEF, was an independent predictor of mortality in the whole group of patients. The three groups presented no significant difference in mortality; however, individuals with reduced LVEF had slightly higher mortality compared to those with mid-range or preserved LVEF (41%, 38%, and 39%, respectively).^11^ Corroborating these findings, Sengelov et al.^12^ showed in an echocardiographic analysis of more than one thousand subjects that GLS was the main predictor of mortality in HF and reduced LVEF patients. Even after adjustment for several variables, such as age, gender, cholesterol, blood pressure, heart rate, ischemic cardiomyopathy, and conventional echocardiographic parameters, no other echocardiographic parameter remained an independent predictor. Therefore, despite the need for further randomized trials to confirm the applicability of the method in clinical practice, the evidence points to the superiority of GLS in predicting the mortality of HF patients - higher than even LVEF.

In this issue of the Journal of Brazilian Society of Cardiology, Maia et al.^13^ conducted a cross-sectional study to verify the correlation between GLS findings and CPET parameters in a sample comprising 26 HF patients of both genders, sedentary, with New York Heart Association (NYHA) functional class II and III, reduced LVEF, and mean age of 47 years. The patients showed a mean strain of -7.5 ± 3.92, maxVO_2_ of 19.09 ± 9.52 mL.kg.min, VE/VCO_2_ slope of 39.43 ± 9.91, HRR of 19.65 ± 17.42, and T_1/2_VO_2_ (s) of 168.61 ± 43.90. They found a statistically significant correlation between GLS and all CPET variables analyzed: HRR, maxVO_2_, VE/VCO_2_ slope, and T_1/2_VO_2_ (s).

Regarding HRR in the first minute post-exercise, patients with slower heart rate reduction showed a strong correlation with lower GLS values. When compared to data collected from CPET, LVEF presented a significant correlation only with maxVO_2_ (direct) and T_1/2_VO_2_ (s) (inverse). On the other hand, GLS was able to predict all variables analyzed by CPET. In short, the study aimed to show the correlation of functional capacity and other CPET variables with GLS, both with established prognostic roles, and that GLS might be more accurate when classifying the severity of HF patients compared to LVEF, providing important knowledge and possible future applications in this scenario.

Nevertheless, the study by Maia et al.^13^ has important issues that should be addressed. The low sample size is a significant limitation of the study, making it impossible to extrapolate the data and use them routinely in clinical practice. Also, the study was not designed and did not have the power to demonstrate the prognostic impact of the findings. On the other hand, the data corroborate previous findings of the literature, indicating that the smaller the GLS value found, the poorer the functional capacity of the individual tends to be; these data are relevant, as they predict a worse prognosis. These outcomes help open new doors and perspectives for further studies in this field, which could confirm important messages conveyed in the literature and strengthened by Brazilian authors.
